# Landscape Setting Drives the Microbial Eukaryotic Community Structure in Four Swedish Mountain Lakes over the Holocene

**DOI:** 10.3390/microorganisms9020355

**Published:** 2021-02-11

**Authors:** Eric Capo, Sofia Ninnes, Isabelle Domaizon, Stefan Bertilsson, Christian Bigler, Xiao-Ru Wang, Richard Bindler, Johan Rydberg

**Affiliations:** 1 Department of Ecology and Environmental Science, Umeå University, 90187 Umeå, Sweden; sofia.ninnes@umu.se (S.N.); christian.bigler@umu.se (C.B.); xiao-ru.wang@umu.se (X.-R.W.); richard.bindler@umu.se (R.B.); johan.rydberg@umu.se (J.R.); 2 UMR CARRTEL, INRAE, Université Savoie Mont Blanc, 74200 Thonon les Bains, France; isabelle.domaizon@inrae.fr; 3 Department of Aquatic Sciences and Assessment, SLU, 75007 Uppsala, Sweden; stefan.bertilsson@slu.se

**Keywords:** sedimentary DNA, 18S metabarcoding, microbial eukaryotes, lakes, Holocene

## Abstract

On the annual and interannual scales, lake microbial communities are known to be heavily influenced by environmental conditions both in the lake and in its terrestrial surroundings. However, the influence of landscape setting and environmental change on shaping these communities over a longer (millennial) timescale is rarely studied. Here, we applied an 18S metabarcoding approach to DNA preserved in Holocene sediment records from two pairs of co-located Swedish mountain lakes. Our data revealed that the microbial eukaryotic communities were strongly influenced by catchment characteristics rather than location. More precisely, the microbial communities from the two bedrock lakes were largely dominated by unclassified Alveolata, while the peatland lakes showed a more diverse microbial community, with Ciliophora, Chlorophyta and Chytrids among the more predominant groups. Furthermore, for the two bedrock-dominated lakes—where the oldest DNA samples are dated to only a few hundred years after the lake formation—certain Alveolata, Chlorophytes, Stramenopiles and Rhizaria taxa were found prevalent throughout all the sediment profiles. Our work highlights the importance of species sorting due to landscape setting and the persistence of microbial eukaryotic diversity over millennial timescales in shaping modern lake microbial communities.

## 1. Introduction

Microbial communities form the base of aquatic food webs and, therefore, influence vital ecosystem functions in aquatic systems, e.g., oxygen production and organic matter cycling [1,2]. The diversity (distribution of species in different lineages) and structure (composition and abundance of species) of these microbial communities are influenced by the environmental conditions, including temperature and nutrient availability [3,4,5]. Consequently, these microbial communities may respond stronger to ongoing climate changes in lakes from boreal, alpine and arctic regions known as being sensitive to climatic stressors [6,7,8]. However, because terrestrial and aquatic ecosystems have also been exposed to, e.g., changes in vegetation and land use [9,10], anthropogenic acidification [11,12] and anthropogenic eutrophication [13,14], it is sometimes difficult to isolate the direct causes of observed changes in lake microbial communities.

Long-term time-series obtained in palaeoecological studies are particularly valuable for investigating the natural responses of microbial communities to environmental and climate changes, because they provide us with a baseline to understand how—and to what extent—aquatic ecosystems are impacted by superimposed human-induced stressors [15,16]. Although the proxies classically used in paleoecology, such as macro- and microscopic remains (including diatoms, cysts and spores), provide key knowledge about the long-term development of the diversity and composition of specific biological groups [16,17,18], such approaches are only applicable to species with identifiable remains, with the remaining biological diversity out of reach. Recent improvements in the DNA sequencing capacity and a deeper understanding of the authentication of genetic signals in sedimentary archives [19,20] make the DNA record preserved in sediments an excellent tool for establishing a baseline for the temporal variability in both community diversity and structure. Over the past decade, DNA metabarcoding has been successfully used in a paleolimnological context to study the past dynamics of both communities of microbial eukaryotes and cyanobacteria [13,21,22,23,24].

Studies of temporal changes in communities of microbial eukaryotes are of special interest because of the numerous ecological roles carried out by such aquatic microbes; for example, intense predation on bacteria, parasitic interactions with fish [25,26] or photosynthesis by phytoplankton [27,28]. In a previous study, Capo et al. [29] illustrated the magnitude of modifications of lake microbial eukaryotic communities over the past 2000 years in relation to global climate changes, such as the Medieval Warm Period, the Little Ice Age and current warming. While past climate changes impacted the aquatic biota in both a large peri-alpine and a small arctic lake, the effects of eutrophication on the peri-alpine lake were stronger and partly overprinted the influence of climate forcing [13,29]. Despite this large potential of studying ancient DNA in sediment archives, there are only a few time series of aquatic microbial eukaryotic communities covering the entire Holocene [30,31] and none concerning boreal or alpine lakes.

The overall objective of the present study was to investigate the long-term (centennial to millennial) natural dynamics in lake microbial eukaryotic community diversity and structure in relation to potential environmental drivers—e.g., climate or nutrient loading. Here, we studied two co-located pairs of Swedish mountain lakes by applying 18S metabarcoding (V7 region) to the DNA extracted from sediment profiles spanning from about 9000 to 200 years before present (yr BP). The lake pairs are located in the mountains of Western Sweden and—except for fish introductions in recent decades (i.e., a time period not included in this study) and low-density reindeer grazing—they have not been exposed to major human perturbations. A fundamental question was if the location (i.e., co-located lakes) or landscape setting (i.e., lakes with similar catchment characteristics: alpine heaths with a thin till and exposed bedrock versus peatland-dominated—hereafter, “bedrock” versus “peatland” lakes) was more important in shaping the diversity and structure of aquatic microbial eukaryotic communities. Furthermore, we aimed to identify the long-term changes in these communities reflecting a response to Holocene climate changes.

## 2. Materials and Methods

### 2.1. Study Sites

**Figure 1:**
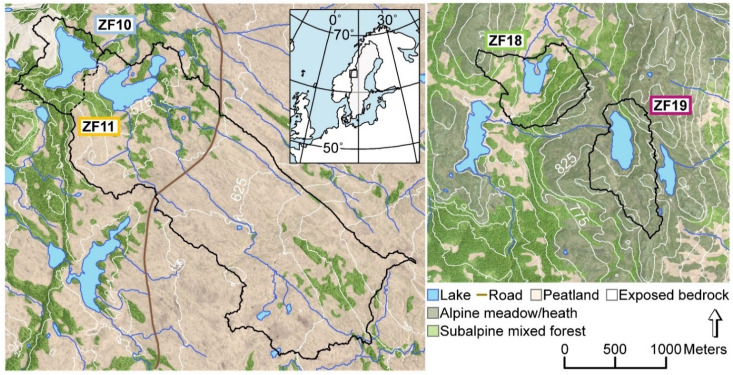
Map of the four study lakes and their catchments, and the location of the study sites in Western Sweden (inset). Black lines delineate the catchment boundaries. ZF10: Bergtjärnen, ZF11: Vargtjärnen, ZF18: Hästskotjärnen and ZF19: Högdalstjärnen.

We studied two pairs of mountain lakes (570–810 m a.s.l.) located in the central part of the Scandes Mountains in the county of Jämtland (Sweden) (Table 1). One co-located pair of lakes, Bergtjärnen (ZF10) and Vargtjärnen (ZF11), are situated on Skalstugufjället (Skalstugu Mountain) 2 km east of the Swedish-Norwegian border (Figure 1). The upper lake, ZF10, has a relatively small and steep catchment underlain predominantly by greywacke (www.sgu.se). Exposed bedrock is found to the west of the lake, while the remainder of the catchment is covered by a thin layer of till. The vegetation cover is mainly subalpine open forest dominated by a mixture of mountain birch (*Betula pubescens* L.) and Norway spruce (*Picea abies* L.). ZF10 drains into Vargtjärnen (ZF11), which has a much larger catchment that also extends to the southeast of the lake, where the bedrock consists of mafic amphibolite and sandstone. The catchment is dominated by blanket peatlands with patches of mixed subalpine open forest along the elevated ridges. The other co-located pair of lakes, Hästskotjärnen (ZF18) and Högdalstjärnen (ZF19), are located 50 km to the southeast of ZF10 and ZF11, on Hammerdalsfjället (Hammardal Mountain). The bedrock in this area consists of quartz- and feldspar-dominated rhyolite, which is largely overlain by till (www.sgu.se (accessed on 7 January 2021)). ZF18 and ZF19 have similar lake areas and catchment sizes, but while the catchment of ZF18 is dominated by peatlands and mixed subalpine open forest, similar to ZF11, the catchment of ZF19 mainly consists of alpine meadows and heaths (with some scattered trees) and areas of exposed bedrock. For simplicity in the text, we refer to the two peatland-dominated lakes (ZF18 and ZF11) as “peatland lakes” and the other two lakes with open subalpine forest, heath and areas of exposed bedrock (ZF19 and ZF10) as “bedrock lakes”. Based on sampling in 2017, the water of the four lakes had circumneutral pH (varying from 6.1 to 6.8), and the concentrations of dissolved organic carbon (DOC) were 2.1, 5.8, 4.4 and 3.1 mg L^−1^ in ZF10, ZF11, ZF18 and ZF19, respectively [32].

### 2.2. Sediment Sampling

In 2018, we collected sediment profiles from each lake using a Russian peat corer (1-m length and 8-cm diameter). Working from the ice surface, a series of overlapping cores (20-cm overlap) were collected in parallel drives about 1 m apart. The individual cores were merged into a continuous profile based on a matching of visual features (such as color changes) and geochemical composition in the overlapping sections. In the field, the sediment cores were first wrapped in plastic and then in aluminum foil and stored at 4 °C until subsampling. Subsampling was performed in the laboratory two weeks after sampling. We collected 2-cm-thick subsamples from the cores for DNA extraction using sterilized plastic spoons. Two subsamples were 1-cm-thick: one subsample from the ZF10 sediment (dated to c. 8295 years before present; yr BP) and one subsample associated with a distinct clay layer in ZF11 (dated c. 4410 yr BP). Approximately 20 subsamples were taken from each sediment profile, yielding 81 samples in total, which resulted in sampling intervals of 4 cm in ZF10 and ZF19 and 8 cm in ZF11 and ZF18. All sediment samples for DNA analysis were then stored untreated at −20 °C until DNA extraction. After removing subsamples for DNA extraction, we collected volumetrically determined samples at 2-cm intervals from the same cores for geochemical analyses. These subsamples were freeze-dried and ground before further analyses.

### 2.3. Dating and Chronology

The sediment profiles were dated using a combination of AMS (accelerated mass spectroscopy) radiocarbon dating (^14^C; Supplementary Table S1) and well-established lead (Pb) stratigraphic age markers (1970 CE and 1100 CE [33]). Lead concentrations were analyzed on 0.05- or 0.2-g powdered sediment using a Bruker S8-Tiger wavelength-dispersive X-ray fluorescence spectrometer (www.bruker.com (accessed on 7 January 2021)) following either the method described by Rydberg [34] (sample sizes of 0.2 g) or a modification of this calibration method adapted for smaller sample sizes (0.05 g). All ^14^C ages were obtained from terrestrial macrofossils and dated at the Radiocarbon Laboratory, Uppsala University, Uppsala, Sweden. Radiocarbon ages were calibrated to calendar years (cal. BP) using the IntCal13 calibration curve [35], and we developed age-depth models (Supplementary Figure S1) using CLAM 2.3 [36] in the statistical platform “R” (v.3.5.2) using a smooth spline (type 4, smooth 0.3). In lakes ZF10 and ZF19, there is a near-linear relationship between age and depth throughout the profile, while, in ZF11 and ZF18, the sedimentation rate was higher in the initial part of the profile (Supplementary Figure S1). The estimated ages of the oldest (deepest) samples used for DNA extraction are 8295, 7330, 7850 and 8430 yr BP, respectively.

### 2.4. Lake Water TOC and Sediment Chlorophyll

To provide some insights into water-quality changes and their potential relationship to changes in the microbial community, we used visible near-infrared spectroscopy (VNIRS) to infer both lake water total organic carbon concentrations at the time when a certain sediment layer was formed (LW-TOC; mg TOC L^−1^ [37]) and sediment chlorophyll concentrations (mg g^−1^ [38]). In brief, VNIR spectra were measured on a FOSS XDS Rapid Content Analyzer (www.fossanalytics.com (accessed on 7 January 2021)) operated in diffuse reflectance mode at a spectral resolution of 0.5 nm (400–2500 nm). Lake water TOC was inferred using the model described by Meyer-Jacob et al. [37], which covers a LW-TOC range from 0.5 to 41 mg L^−1^ and has a cross-validated R^2^ of 0.57 and a prediction error of 4.4 mg L^−1^. Sediment chlorophyll (i.e., chlorophyll *a*, its derivatives and their respective degradation products) was inferred using the calibration model of Michelutti et al. [38] using the absorbance peak area between 650 and 700 nm. The model has a reported R^2^ of 0.72 (*p* < 0.05) and an estimated lowest limit of detection of 0.01 mg g^−1^.

### 2.5. DNA Analysis

All laboratory procedures relating to the DNA analysis were carried out in a dedicated, clean DNA laboratory and followed strict protocols to ensure analytical quality, i.e., working under sterile conditions, using sterile disposable labware and storing subsamples and DNA extracts at −20 °C. We included blank controls (i.e., opened containers with ultrapure water) in each step of the process: 9 blanks collected during the subsampling, 8 blanks collected during DNA extraction and 4 blanks collected during PCR assays (21 blanks in total). DNA extraction was made on ~0.5 g of wet sediment using the DNeasy Powersoil kit according to the manufacturer’s instructions (Qiagen, Carlsbad, CA, USA). DNA extract from each sample (~25 ng) was then used to amplify via PCR the V7 region of the 18S rRNA gene by targeting a 260-bp-long fragment using the general eukaryotic primers 960f (5’-GGCTTAATTTGACTCAACRCG-3’ [39]) and NSR1438 (5’-GGGCATCACAGACCTGTTAT-3’ [40]). Each PCR was performed in a total volume of 25 µL containing 3 µL of 10x NH4 reaction buffer, 1.2 µL of 50-mM MgCl_2_, 0.25 µL of BioTaq (Bioline), 0.24 µL of 10-mM dNTP (deoxyribonucleotide triphosphate), 0.36 µL of 0.5-µg/µL BSA (Bovine Serum Albumin) and 1 µL of each primer (500 nM). The amplification conditions consisted of an initial denaturation at 94 °C for 10 min, followed by 35 cycles of 1 min at 94 °C, 1 min at 55 °C and 1 min 30 s at 72 °C and terminated by a final 10-min extension at 72 °C. Sample-specific molecular barcode combinations for the forward and reverse primers were performed to reduce the frequency of analytical cross-contamination between PCR products [41]. To assess the consistency of the PCR amplification and DNA sequencing, DNA extracts from two of the ZF10 sediment samples were PCR-amplified and sequenced in duplicate. The 104 molecular inventories (81 sediment samples + 2 PCR replicates + 21 blanks) were multiplexed using 104 of the 225 possible molecular barcode combinations with 15 forward and 15 reverse tagged primers. The PCR products obtained from the DNA extracted from each sample were quantified using a Quant-it PicoGreen kit (Invitrogen, Carlsbad, CA, USA), pooled in equimolar amounts and purified using the QIAquick PCR Purification kit (Qiagen, Carlsbad, CA, USA). Library preparations (Illumina TruSeq PCR-free) and paired-end (2 × 250 bp) sequencing (MiSeq Illumina instrument) were performed at SciLifeLab (NGI, Stockholm, Sweden).

### 2.6. Bioinformatics

Raw sequence reads were deposited in FigShare (https://doi.org/10.6084/m9.figshare.11376672.v1 (accessed on 7 January 2021)) and were analyzed using the procedure outlined in Capo et al. [29]. Briefly, the paired-end reads were merged and cleaned (no undefined bases, minimum sequence length of 200 bp, no sequencing error in primers and removing of chimera) using the UPARSE and UCHIME tools [42,43], allowing to obtain a total of 5,847,791 DNA sequence reads (hereafter, DNA reads) distributed across the 104 molecular inventories that were subsequently grouped into 4050 OTUs (Operational Taxonomic Units) with 95% nucleic acid identity as the threshold. To delineate the similarity threshold, we followed the recommendations from Mangot et al. [44], who estimated the errors during PCR and sequencing by introducing an internal 18S standard (a unique alien DNA sequence) and demonstrated that 95% was a relevant cut-off threshold for OTU delineation, allowing to limit the artificial inflation of the number of OTUs In addition, we verified this threshold by sequencing a mock community in the same experimental conditions (primers, sequencing platform and bioinformatics pipelines); as reported in Capo et al. [29], 16 OTUs from the 16 species of the mock community were obtained when applying a 95% threshold.

The taxonomic annotation was performed by BLAST against the SSURef SILVA database NR108 [45] that was extended with lacustrine DNA reads originating from previous studies [44,46,47,48]. This database is provided with the PANAM2 pipeline (https://github.com/panammeb/PANAM2 (accessed on 7 January 2021)). For blank corrections, we removed a total of 1326 OTUs seen at relative abundances >10% higher in the blanks than in molecular inventories from sediment samples. We removed all the OTUs affiliated to metazoans, embryophytes, fungi (except chytrids), Cercozoa nuclear, Cryptophyta nucleomorph and unclassified eukaryota in order to consider only microbial eukaryotes in our analysis (Supplementary Table S2). Rarefaction curves were performed for the 83 molecular inventories (81 sediment samples + 2 PCR replicates) using the function “rarecurve” from the vegan R package [49]. To make the analyses more robust, we also removed the OTUs represented by less than 4 DNA reads in the entire dataset. Additionally, 9 of the sediment samples gave molecular inventories with a low number of DNA reads (<23,500 reads) and were removed. The 74 resulting molecular inventories were rarefied at 23,500 reads.

To evaluate the similarity between analytical replicates, we performed a Bray-Curtis dissimilarity-based hierarchical clustering analysis on the molecular inventories from the 16 sediment samples and two PCR replicates from ZF10 using “vegdist” and “hclust.order” functions from the vegan R package [49]. Our outputs showed that the replicates clustered together and had lower Bray-Curtis dissimilarity values (0.26 and 0.18 for the two PCR replicate samples from the sediment samples with IDs 18 and 20, respectively) as compared to the average between-sample difference for the other sediment samples (Supplementary Figure S2a). The PCR replicates were not used for any further analyses of the data. In addition, we removed the sample at the base of the sediment record from Lake ZF10, because this sample, consisting only of glacial clay, likely corresponds to a time prior to lake formation (Supplementary Figure S3). The final dataset thus includes molecular inventories for 71 sediment samples from the four lakes.

### 2.7. Data Analysis

We used the rarefied OTU abundance table to calculate the OTU richness, Shannon diversity index and Simpson dominance index. Using the vegan R package [49], we calculated the Bray-Curtis dissimilarity indices and performed nonmetric multidimensional scaling (nMDS) and hierarchical clustering analyses (“metaMDS” and “hclust” functions, vegan R package) [49]. To evaluate the differences in community structures between the four lakes, ANOSIM analysis was performed using PAST [50]. Venn-diagrams showing the number of OTUs specific to single lakes and those shared between lakes were drawn using the software Venny 2.1 [51]. In order to investigate the relationships between the structure of the microbial eukaryotic community, LW-TOC and sediment chlorophyll concentrations in a certain sample, we employed the function “env.fit” from the vegan R package [49] based on the goodness-of-fit statistics (defined as r^2^ = 1-ssw/sst, where ssw and sst are the within-group and total sums of squares, respectively). Bivariate correlations were calculated between the diversity metrics obtained from the molecular inventories, LW-TOC and sediment chlorophyll concentrations as the Pearson coefficient correlations. The significance level was set to 0.05.

## 3. Results

### 3.1. Past Diversity of the Microbial Eukaryotic Communities

In the 71 sediment samples, we identified a total of 1721 OTUs (defined at the 95% nucleic acid identity) distributed across 41 microbial eukaryotic groups (Table 2). The 12 most dominant microbial eukaryotic groups accounted for 95% of the DNA reads and most of the OTUs (1424 OTUs) (Table 2). The 12 groups included strict heterotrophs (Apicomplexa, Cercozoa, Ciliophora, Chytrids and Perkinsea); phototrophs (Bacillariophyta and Chlorophyta) and groups containing heterotroph, phototroph and mixotroph taxa, such as Dinophyceae and Chrysophyceae (Table 2). The 20 most abundant OTUs together accounted for more than 62% of the total number of DNA reads. The rarefaction curves indicated that the microbial diversity in ZF11 and ZF18 were analyzed to saturation, while it was incompletely described in ZF10 and ZF19 (Supplementary Figure S2b).

### 3.2. Long-Term Temporal Dynamics in Microbial Eukaryotic Communities

**Figure 2:**
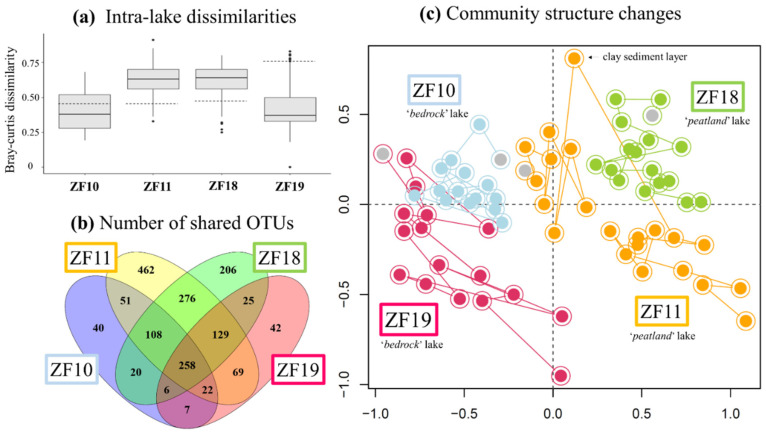
(**a**) Boxplots showing the amplitude of changes between depths (ages) for each lake in terms of the Bray-Curtis dissimilarities. Hatched bars correspond to the Bray-Curtis dissimilarity values measured between the oldest sediment sample and the second-oldest sediment sample for each sediment core. (**b**) Venn-diagram illustrating the numbers of Operational Taxonomic Units (OTUs) common to the four lakes, between each pair of lakes and specific to each lake. (**c**) Temporal changes in the microbial eukaryotic community in sediments from the four lakes. The nonmetric multidimensional scaling (nMDS) is based on the Bray-Curtis dissimilarity values calculated from the table of standardized OTU relative abundances. The colors denote the respective lake. The oldest sediment samples from each time series are highlighted in gray. The position of the molecular inventory corresponding to the clay layer in the ZF11 sediment profile was displayed in the nMDS plot.

**Figure 3:**
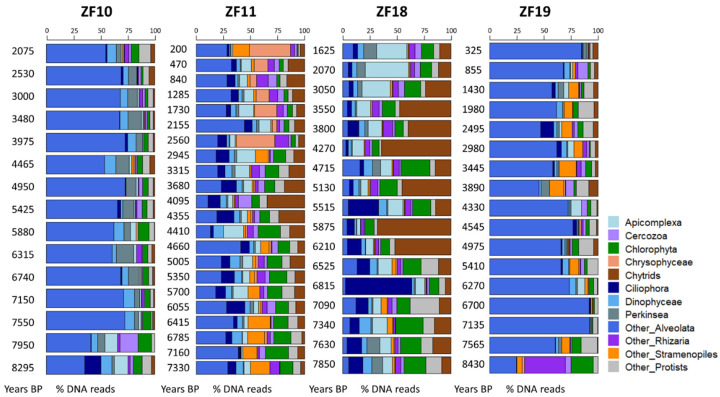
Bar plots showing the temporal changes in the relative abundance of the microbial eukaryotic groups for each lake. For each molecular inventory, the sum of DNA reads for the 12 groups is set to 100%. BP: before present.

The time series revealed a higher temporal variability in the microbial eukaryotic community structure along the sediment profiles from the two peatland lakes (ZF11 and ZF18) compared to those from the two bedrock lakes (ZF10 and ZF19), as indicated by lower Bray-Curtis dissimilarities for the bedrock lakes (Figure 2a). Temporal changes in relative abundance—i.e., based on the number of DNA reads—of the microbial eukaryotic groups were observed in all sedimentary records and, more particularly, the two peatland lakes (Figure 3). In ZF11, there was a decrease in Stramenopiles after 4660 yr BP and an increase in Chrysophyceae from c. 2560 yr BP. In ZF18, a noticeable increase was detected in Ciliophora at c. 6815 yr BP, as well as in Apicomplexa and Cercozoa from c. 3050 yr BP.

In the two bedrock lakes, the oldest analyzed sediment samples (c. 8295 yr BP in ZF10 and c. 8430 yr BP in ZF19) showed a different microbial community as compared to the next analyzed sample in the sequence. In ZF10, Ciliophora was the second-most abundant group in the oldest sediment sample (>14% of DNA reads), whereas it is virtually absent in the sample above and comprises less than 3% of the DNA reads throughout the remainder of the core. In ZF19, Rhizaria disappeared almost completely between the two oldest sediment samples in the profile (>37% and <1% relative abundance for the oldest and second-oldest sediment samples, respectively) and comprised less than 5% in the remainder of the sediment profiles (Figure 3).

**Figure 4:**
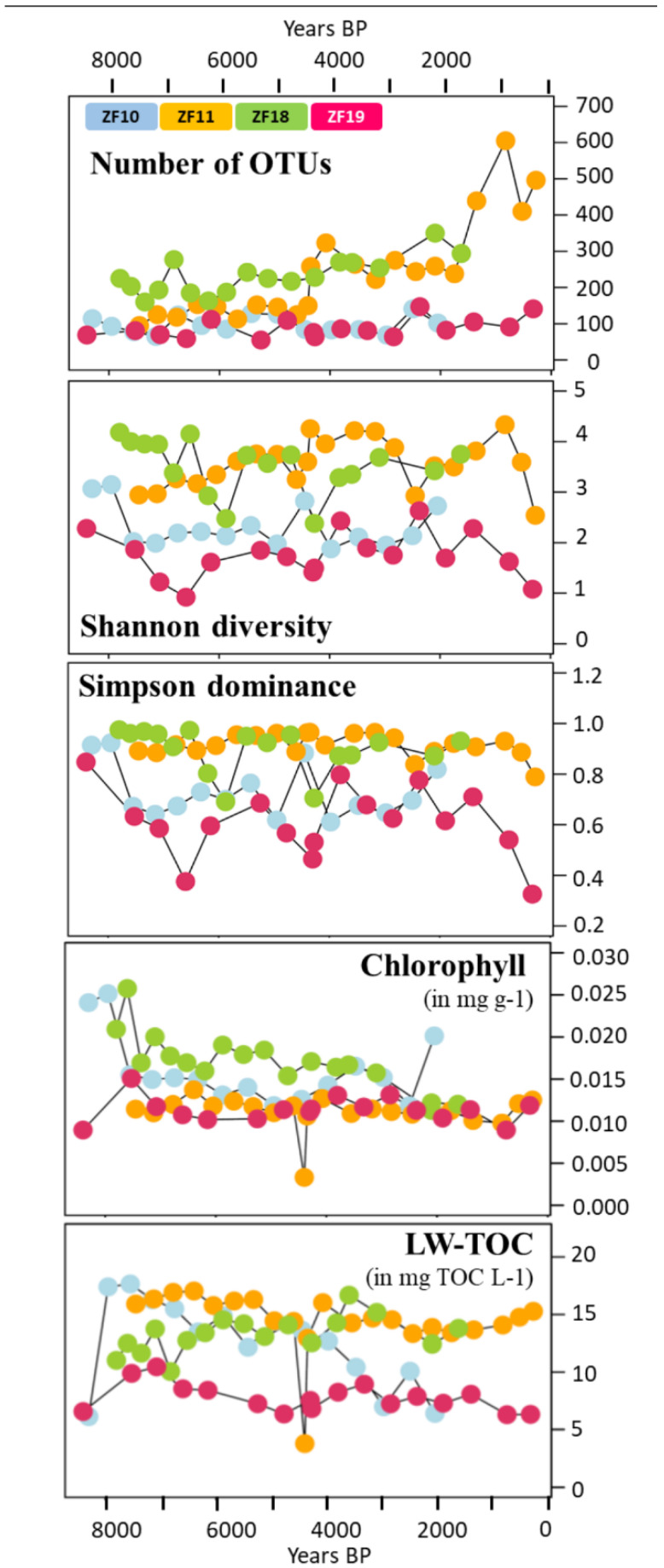
Time series of the diversity metrics of the microbial eukaryotic communities (OTU richness, Shannon diversity index and Simpson dominance index); sediment chlorophyll concentration (mg g^−1^) and LW-TOC (mg TOC L^−1^). The four colors correspond to the four lakes, as indicated in the uppermost plot. The sample corresponding to the clay layer in the ZF11 sediment profile is the one with the lowest sediment chlorophyll concentration and LW-TOC values.

In general, the sediment profiles from the two bedrock lakes had a lower OTU richness throughout their profiles compared to the peatland lakes. The number of OTUs in the sediment samples from the bedrock lakes varied from 55 to 147 for each individual sample, without any temporal trend (Figure 4). In contrast, the sediment profiles from the two peatland lakes increased in the number of OTUs with the decreasing sediment depth and age. In ZF11, the OTU richness per sediment sample started at ~150, increased to >250 after c. 4660 yr BP and then increased further after 2155 yr BP to a peak of 605 OTUs at 840 yr BP. In ZF18, the OTU richness displayed a steady increase from ~200 to 250 at the bottom of the profile to ~300 at the top of the profile (Figure 4). The Shannon diversity index was lower throughout the sediment profiles of the two bedrock lakes (0.92–3.15) compared to the peatland lakes (2.38–4.33), and similar patterns were found for the Simpson dominance index (Figure 4).

**Figure 5:**
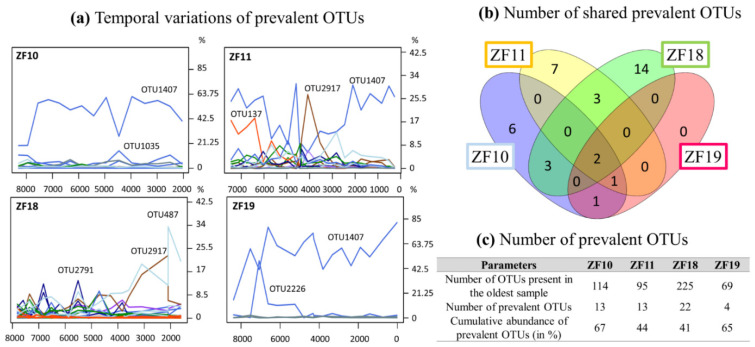
(**a**) Temporal changes in the relative abundance of the prevalent OTUs for each lake. This number of DNA reads from each prevalent OTU corresponds to their relative abundance for each sample rarefied at 23,500 DNA reads. (**b**) Number of shared prevalent OTUs. (**c**) Number of prevalent OTUs.

For each lake, certain OTUs were present throughout the sediment profile, which we hereafter refer to as “prevalent OTUs” (Figure 5a–c and Supplementary Table S3). Two such prevalent OTUs (OTU3208 Perkinsea and OTU1407 unclassified Alveolata) were detected in all sediment samples from all four lakes (Figure 5b and Supplementary Table S3). In the two bedrock lakes, OTU2226 (unclassified Alveolata) was also detected as a prevalent OTU. In ZF19, only one other prevalent OTU, a Perkinsea (OTU977), was detected. In contrast, >10 prevalent OTUs were detected in each of the other three lakes, including Chlorophytes, Stramenopiles and Rhizaria OTUs (Figure 5c and Supplementary Table S3).

### 3.3. Landscape Setting and Microbial Eukaryotic Communities

The microbial eukaryotic communities in the bedrock lakes were dominated by taxa from unclassified Alveolata, Dinophyceae and Chlorophyta groups (Figure 3 and Table 2). In contrast, the dominant microbial groups in the peatland lakes were Chlorophytes, Chytrids, Ciliophora and Apicomplexa, as well as unclassified members of Alveolata and Stramenopiles (Figure 3 and Table 2). Overall, 44% of all OTUs were lake-specific, with the two peatland lakes having a higher number of lake-specific OTUs (462 and 206) compared to the bedrock lakes (40 and 42; Figure 2b). The sediment samples from the two peatland lakes had the highest number of shared OTUs, with 771 being common for these two lakes, including 276 that were only found in sediment samples from the peatland lakes. There was also a higher OTU richness in the sediment samples from the peatland lakes, with a total number of 1375 and 1028 OTUs found within the ZF11 and ZF18 profiles compared to the 512 and 558 OTUs found in the sediment profiles from the bedrock lakes (ZF10 and ZF19), respectively (Table 2). The nMDS analysis showed that the between-lake differences in the structures of the microbial eukaryotic communities seem to be based on differences in the landscape setting rather than geographic location (Figure 2c). The bedrock lakes (ZF10 and ZF19) plot on the left-hand side of the nMDS graph, while the peatland lakes (ZF11 and ZF18) plot on the right-hand side (Figure 2c); this dissimilarity is supported by the ANOSIM analysis (*p* < 0.001).

We observed temporal correlations between the community structure in the two peatland lakes (ZF11 and ZF18) and the inferred LW-TOC and sediment chlorophyll concentrations, as well as between the ZF19 community structure and LW-TOC concentrations (Table 3). It should be noted that the very low LW-TOC and sediment chlorophyll concentrations in one ZF11 sediment sample occurred in a distinct 1-cm-thick clay layer (c. 4410 yr BP) (Figure 4). This clay layer also corresponded to the shift observed in the ZF11 community structure (Figure 2c). In ZF10, the Shannon diversity and Simpson dominance indices correlated with the sediment chlorophyll concentrations (r = 0.75 and r = 0.69, respectively; *p* < 0.001) (Supplementary Table S4, whereas we found no correlations between the microbial community structure and the two inferred VNIRS parameters (Table 3). The sediment chlorophyll concentrations in ZF10 were also strongly negatively correlated with the relative abundance of the “Other Alveolata” (r = −0.81, *p* < 0.001; Supplementary Table S4). For ZF18, the increase in OTU richness and Apicomplexa relative abundance at c. 3050 yr BP correlated with a decrease in the sediment chlorophyll concentrations (Figure 3 and Figure 4 and Supplementary Table S4).

## 4. Discussion

### 4.1. Landscape Setting, Not Climate Shapes Lake Microbial Eukaryotic Communities

Based on the DNA preserved in lake sediments, our study revealed larger similarities in the microbial eukaryotic communities between lakes with a similar landscape setting. This implies that landscape setting had a larger influence on shaping the structure of these four lake’s microbial eukaryotic communities than location. Similar patterns between the diversity and structure of aquatic microbial eukaryotic communities and environmental conditions (e.g., DOC/nutrient concentrations) have been shown in several short-term studies [52,53,54,55,56]. Previous studies have also described the microbial eukaryotic diversity in lakes [57,58], but it might be difficult to assess the slow and gradual process that shapes microbial communities using the snapshot view provided when looking at the chemistry and biology in lake water samples. Here, our study shows that catchment characteristics and landscape setting are also important in the long-term perspective needed to truly understand the temporal changes in lake ecosystems.

In terms of relative abundance (Figure 3), the two bedrock lakes are both highly dominated by unclassified Alveolata, while the peatland lakes have a more diverse microbial community, where groups such as Ciliophora, Chlorophyta and Chytrids are found in high relative abundance. Other works have shown that a decreased input of organic matter from the catchment to the lake can cause a community shift towards diatoms, Chrysophytes, Cryptophytes and Dinophytes [5,13,59,60]. We did not observe any such shift in these major plankton groups in the sediment profiles of the bedrock lakes, which are characterized by relatively low DOC concentrations (2.1 and 3.1 mg L^−1^). In the peatland lakes a weak correlation was found between the LW-TOC concentrations (i.e., a proxy for the allochthonous input of organic matter) and the microbial communities (Table 3).

Our data suggest that lakes in the same region with a similar landscape setting could be expected to have a comparable microbial community. However, when looking at the OTU level, less than half of the identified OTUs are shared between the lake pairs (17% and 45% for the bedrock and peatland lakes, respectively). The fact that the microbial communities are very lake-specific and that there are large between-lake variations is consistent with earlier studies that have reported that intraregional environmental variability has an effect on the diversity and structure of aquatic microbial communities [52,54,55]. Here, such patterns also appeared, with a higher number of shared OTUs in the lakes with similar landscape settings compared to adjacent lakes. On the other hand, the number of OTUs found in ZF10 that are not found in the downstream lake ZF11 is lower (4%) as compared to the number of OTUs found in ZF19 but not in ZF18 (8%), which indicates that the hydrological connection does have an effect on the distribution of microbial eukaryotes between interconnected lakes. Together these spatial patterns indicate that we cannot expect the microbial community to be the same, even in adjacent lakes that are hydrologically connected. This implies that even if a shift in the microbial community—or in a specific biological group—in one lake can be attributed to, e.g., climate change or eutrophication, this does not necessarily mean that the same change in a nearby lake can be attributed to the same driving factor.

Several studies have identified interactions between lake microbial communities and climate, both acting and reacting (for review, see [61]), where microbial eukaryotes appear to be modified in response to long-term climatic fluctuations [29,62,63,64]. In Northern Europe, pollen-based climate reconstructions of temperature indicate that the period from 7000 to 5600 yr BP and, more broadly, during 8000–4000 yr BP, was warmer [65], followed by an overall linear cooling trend starting from c. 4000 yr BP, with relatively warmer periods from this overall cooling trend occurring during 3000–1000 yr BP and the last 150 years. Even if we do find some common features in the temporal variations in the microbial eukaryotic communities within the four lakes (Figure 4 and Figure 5), there does not appear to be any major synchronous changes in the microbial communities that would indicate a common driver such as climate over the studied period. Here, it should be noted that large differences between proxy data on microbial communities and climate (e.g., the very different temporal resolutions and uncertainties) prevented us from performing any meaningful correlation analysis. This said, our data suggest that long-term climate changes do not appear as an obvious driver of the microbial biota of these mountain lake ecosystems.

### 4.2. The Importance of Early Community Structure for Shaping a Lake’s Microbial Eukaryotic Community at Later Dates

Even if the landscape setting is important in shaping the aquatic microbial community (Figure 2c), there are other processes and factors that also have an influence. One such factor is pioneer communities and how they influence the later community structure through legacy effects and species sorting [66,67]. Since we did not analyze the absolute base of each profile and, thus, do not have data on the true pioneer communities in each lake, we looked at the prevalent OTUs found throughout each profile, beginning from c. 7500 to 8500 yr BP (Figure 5). This gave us some insight into the temporal modifications of microbial communities in aquatic systems. Of the OTUs that were identified in the oldest sample from each lake, 11%, 14%, 10% and 4% were prevalent for ZF10, ZF11, ZF18 and ZF19, respectively. In terms of the number of DNA reads, these prevalent OTUs represent between 41% and 67% of all identified DNA reads in each sediment profile (Figure 5c); in ZF10, ZF11 and ZF19, the predominant OTUs detected in the oldest analyzed sediment samples (c. 8430–7330 yr BP) are still the dominant OTUs in the most recent analyzed sediment sample in each respective lake (Figure 5a and Supplementary Table S3). This finding supports the idea that microbial eukaryotes present early in a lake’s history have an influence on the composition of later communities, especially because they were still among the most abundant members in the molecular inventories in the most recent sediment samples that were analyzed.

In relation to the discussion of legacy effects and species sorting, it is interesting to also consider the 1-cm-thick clay layer in the ZF11 record (c. 4410 yr BP). This clearly defined clay layer suggests that an abrupt erosional event occurred in the lake catchment. The sudden transport of large amounts of clay particles to the lake would likely temporarily reduce light penetration in the water column, which would have negatively impacted many light-dependent organisms living in the lake, e.g., phototrophic bacteria and algae, as well as invertebrates and fish that are dependent on these primary producers for survival. During such an event, it is also likely that new species would be transported to the lake from upstream/upslope locations in the catchment. This leads to a kind of reset of the microbial eukaryotic community. This event appears to have had a profound effect on the microbial community, which is notably marked by an abrupt increase in the number of OTUs (Figure 4) and a temporary drop in the relative abundance of the dominating prevalent Alveolata taxa (OTU1407), coupled with an increase of the prevalent chytrid taxa (OTU2917) (Figure 5). With time, OTU1407 returned to being the dominant prevalent OTU (Figure 5), but at the same time, there was no return to the pre-event community structure (Figure 2c). Because OTU1407 is an unclassified alveolate, a microbial superphylum with ecologically various members—from strict phototrophs to strict heterotrophs—we cannot draw any conclusions about the role of this OTU in the microbial community and its link with the environmental drivers. Taken together, our findings suggest that both legacy effects and species sorting play important roles in shaping the present-day microbial community in ZF11.

### 4.3. Reliability of the Sedimentary DNA Signal to Unravel Past Lake Microbial Diversity

Although previous studies have highlighted that molecular signals extracted from sedimentary archives accurately represent past aquatic microbial communities [20,68,69,70,71], there is a need to discuss the possibility that a part of the molecular microbial signal we obtained in our sediment records can originate from catchment DNA or microbial eukaryotes that are indigenous to the sediment. In our case, an input of catchment DNA could explain the strong differences in the sediment microbial eukaryotic communities seen in the two bedrock lakes as compared to the two peatland lakes. For instance, Giguet-Covex et al. [72] revealed that the transfer of catchment DNA occurs through the isolation of DNA originating from terrestrial plants or mammals in sediment records, but it is more difficult to track the origin (aquatic or terrestrial) of microbial DNA. Previous studies made on water samples revealed that some of the microbes—both prokaryotes and eukaryotes—living in freshwater systems originated from soils from the surrounding catchments [73,74]. Microbial eukaryotes seem to be more restricted; for example, whereas Crump et al. [73] found that 58% and 43% of the bacteria and archaeal taxa from soils could be detected in lake water, only 18% of the soil microbial eukaryotes were found in the same freshwater systems. Data from Grossman et al. [57] showed the predominance of certain groups of microbial eukaryotes in soils—including Cercozoa, Amoebozoa and Apicomplexa—while other phyla such as Ciliophora, Chrysophyceae, Dinophyceae, Synurophyceae, Chytrids and Diatoms were mostly found to be abundant in freshwater biomes. Based on this, the most abundant microbial eukaryotic groups detected in the sediment profiles of our four lakes appear to have an aquatic origin. The exception might be Apicomplexa, where some studies have suggested that it is only found in very low abundance in lake water [57]. However, others have described members of Apicomplexa as freshwater parasites [75,76], and their relatively high abundance in all four sediment profiles suggests that their origin is the water column. It is noticeable that the molecular signal from certain microbial eukaryotic groups with an aquatic origin, such as Cryptophytes and Haptophytes, might not be well-preserved in sediments [68].

Several abundant OTUs could not be robustly taxonomically identified using the SILVA database, most likely because they have never been cultivated or characterized by sequencing. The most abundant OTU of the dataset (OTU1407) was, for instance, assigned as an unclassified alveolate, as were three others of the most abundant OTUs (OTU2226, OTU3337 and OTU1035) (Supplementary Table S3). Because the members of Alveolata are ecologically diverse, the taxonomic resolution is too low to infer if its origin is aquatic (water column or sediment) or the terrestrial catchment. Due to the use of relatively short DNA fragments for our analyses (average of 260 bp), there is a limited potential to increase the taxonomic resolution and coupled identification of the microbial eukaryotes to the species level, which limits the conclusions that may be drawn. For instance, Delmont et al. [77] showed that shotgun sequencing data were useful to discriminate between two closely related populations of SAR11 (a ubiquitous clade of marine bacteria) associated with either warm or cold ocean currents. Applying shotgun sequencing—or metagenomics—on sedimentary DNA [78,79,80,81] may remedy some of these limitations in offering an approach to explore the microbial DNA record in sedimentary archives at a finer taxonomic resolution.

## 5. Conclusions

Our findings demonstrate that microbial eukaryotic communities can differ substantially in different lakes, even at very short distances (e.g., hydrologically connected lakes). Additionally, local factors that set adjacent lakes apart, such as landscape setting, episodic events and species sorting, play an important role in the long-term development of the microbial community. Our results also indicate that each lake appears to behave independently. Finally, even if the climate may exert some influence on emergent microbial communities in a specific lake, both the timing and direction of changes in the community structure can be strongly controlled or modified by site-specific conditions such as the aquatic vegetation, food-web structure, water depth or light climate.

## Figures and Tables

**Table 1:** Location and characteristics of the study sites.

Lake	Coordinates	Elevation (m)	Lake Area (ha)	Catchment Area (ha)	Max. Lake Depth (m)	Avg. Catchment Slope (°)
ZF10 (Bergtjärnen)	63°37’27” N	573	14	31	341	16.5
	12°15’35” E					
ZF11 (Vargtjärnen)	63°37’17” N	564	12	454	14	6.6
	12°16’12” E					
ZF18 (Hästskotjärnen)	63°18’52” N	735	6	51	8	12.6
	12°54’11” E					
ZF19 (Högdalstjärnen)	63°18’3” N	812	9	51	14	12.3
	12°55’14” E					

**Table 2:** Number of DNA reads (#DNA-reads) and Operational Taxonomic Units (OTUs) (#OTUs) for each microbial eukaryotic group detected in all samples and for each lake independently. The 12 most dominant microbial eukaryotic groups are highlighted in bold.

Microbial Eukaryotic Groups		All lakes		Lake ZF10		Lake ZF11		Lake ZF18		Lake ZF19	
		#DNA-Reads	#OTUs	#DNA-Reads	#OTUs	#DNA-Reads	#OTUs	#DNA-Reads	#OTUs	#DNA-Reads	#OTUs
Number of Molecular Inventories		72		15		22		17		17	
Alveolata	**Apicomplexa**	114,977	86	10,822	22	41,631	63	54974	47	7550	24
	**Ciliophora**	100,614	196	5918	46	40,639	145	47,189	127	6868	48
	**Dinophyceae**	82,078	135	24,297	51	26,267	106	20,734	89	10,780	49
	**Perkinsea**	60,465	69	28,327	28	7885	54	15,889	42	8364	30
	Voromonas	1944	8	202	5	1346	4	233	5	163	3
	**Other_Alveolata**	643,595	105	220,235	45	136,264	93	26,718	63	260,378	47
Amoebozoa	Centramoebida	68	3	42	1	24	2	2	1	0	0
	Tubulinea	1362	19	28	1	1219	18	101	6	14	3
Cryptophyta	Cryptomonadales	1571	4	473	1	248	3	310	2	540	2
	Cryptophyta_2	608	1	243	1	39	1	325	1	1	1
	Cryptophyta_3	4	1	0	0	4	1	0	0	0	0
	Cryptophyta_4	289	3	2	1	174	1	111	3	2	1
	Pyrenomonadales	1705	7	329	2	1062	5	313	6	1	1
	Other_Cryptophyta	313	7	0	0	46	7	266	2	1	1
Haptophyta	Coccolithales	6	2	0	0	0	0	6	2	0	0
	Pavlovales	113	1	0	0	0	0	113	1	0	0
	Phaeocystales	974	3	15	1	341	3	618	1	0	0
	Prymnesiales	1820	7	458	2	1008	7	350	4	4	1
	Syracosphaerales	2	1	2	1	0	0	0	0	0	0
	Other_Haptophyta	17436	17	6604	12	5557	13	1490	12	3785	10
Opisthokonta	Choanoflagellida	806	8	375	1	254	5	177	4	0	0
	**Chytrids**	183,194	233	7126	58	58,565	191	107,699	145	9804	84
	Opisthokonta_incertae_sedis	954	1	1	1	261	1	690	1	2	1
Rhizaria	**Cercozoa**	56,498	244	10,334	75	23,998	194	10,745	131	11,421	61
	**Other_Rhizaria**	60,868	78	4897	18	24,895	64	17,820	38	13,256	31
Rhodophyta	Bangiophyceae	202	3	0	0	202	3	0	0	0	0
	Florideophyceae	873	7	581	1	235	5	57	2	0	0
Stramenopiles	**Bacillariophyta**	26,914	32	2231	14	6479	25	9207	20	8997	16
	Bicosoecida	21,556	49	1145	12	6678	39	4434	26	9299	15
	**Chrysophyceae**	39,585	44	1532	13	34283	39	1953	28	1817	19
	Dictyochophyceae	63	4	0	0	8	3	53	2	2	1
	Eustigmatophyceae	6828	9	1632	3	489	5	3530	7	1177	2
	Labyrinthulida	940	8	1	1	135	5	730	6	74	2
	MAST (1, 2, 3, 4, 7)	696	15	1	1	528	14	145	7	22	3
	Oomycetes	12,365	38	2346	14	4694	28	3137	24	2188	18
	PX_clade	4751	13	394	7	1544	10	2772	10	41	3
	Synurophyceae	3521	15	4	3	894	9	926	15	1697	5
	**Other_Stramenopiles**	65,265	55	1301	15	33,985	50	7677	30	22,302	15
Viridiplantae	**Chlorophyta**	147,386	147	20,410	43	52,483	126	56,888	94	17605	47
	Streptophyta	5240	40	171	11	2619	30	1105	23	1345	14
	Other_Viridiplantae	51	3	21	1	17	3	13	1	0	0
Total		1,668,500	1721	352,500	512	517,000	1375	399,500	1028	399,500	558

**Table 3:** Temporal correlations between the community structure and inferred LW-TOC and sediment chlorophyll concentrations for each lake.

Parameters	Lake ZF10		Lake ZF11		Lake ZF18		Lake ZF19	
	r	*p*	r	*p*	r	*p*	r	*p*
**chlorophyll**	0.34	0.08	0.64	0.035	0.56	0.004	0.02	0.891
**LW-TOC**	0.31	0.1	0.47	0.031	0.4	0.033	0.38	0.029

## Data Availability

Raw data are available at figshare https://doi.org/10.6084/m9.figshare.11376672.v1.

## Supplementary Materials

The following are available online at https://www.mdpi.com/2076-2607/9/2/355/s1: Supplementary Table S1. AMS radiocarbon-dated samples. Calibrated ages are given as 95% confidence ranges. Supplementary Table S2. List of the OTUs removed based on blank correction and not-targeted organisms. Supplementary Table S3. List of the OTUs, alongside their prevalence in each lake, their total abundance in the molecular inventories, their nucleotide sequences, the outputs of the BLAST for the unclassified taxa and the taxonomic affiliations from the pipeline PANAM2Supplementary Table S4. Pearson correlations between biological parameters and environmental parameters (LW-TOC and chlorophyll) for each lake independently. The r corresponds to the value of the Pearson correlation coefficient and the *p* to the *p*-values obtained. Supplementary Figure S1. Age-depth models for ZF10, ZF11, ZF18 and ZF19 developed with CLAM 2.3, where black lines indicate the best fit and the light grey lines the 95% confidence intervals. Blue represents ^14^C samples and green the ages inferred from Pb concentrations. The full, horizontal, black line near the bottom of the age-depth model for ZF10 indicates the time of lake formation, and the hatched horizontal lines at the bottom of the age-depth models for ZF11, ZF18 and ZF19 indicate the oldest sample for each profile. Supplementary Figure S2. (a) Outputs of the hierarchical clustering tree on molecular inventories from Lake ZF10 showing the cluster formed by the two pairs of PCR replicates (ZF10-RI-66 and ZF10-RI-78). (b) Rarefaction curves of the molecular inventories obtained from the sedimentary archives of the four lakes. Supplementary Figure S3. nMDS plot of the ZF10 molecular inventories, including the sample corresponding to the time before lake formation. The relative abundance of each microbial eukaryotic group before and after lake formation is displayed in the bar plot.
