# Supplementary figures and images for: Landscape Setting Drives the Microbial Eukaryotic Community Structure in Four Swedish Mountain Lakes over the Holocene
Source: Microorganisms. 2021 Feb 11;9(2):355. doi: 10.3390/microorganisms9020355 (PMC7916980; doi:10.3390/microorganisms9020355)

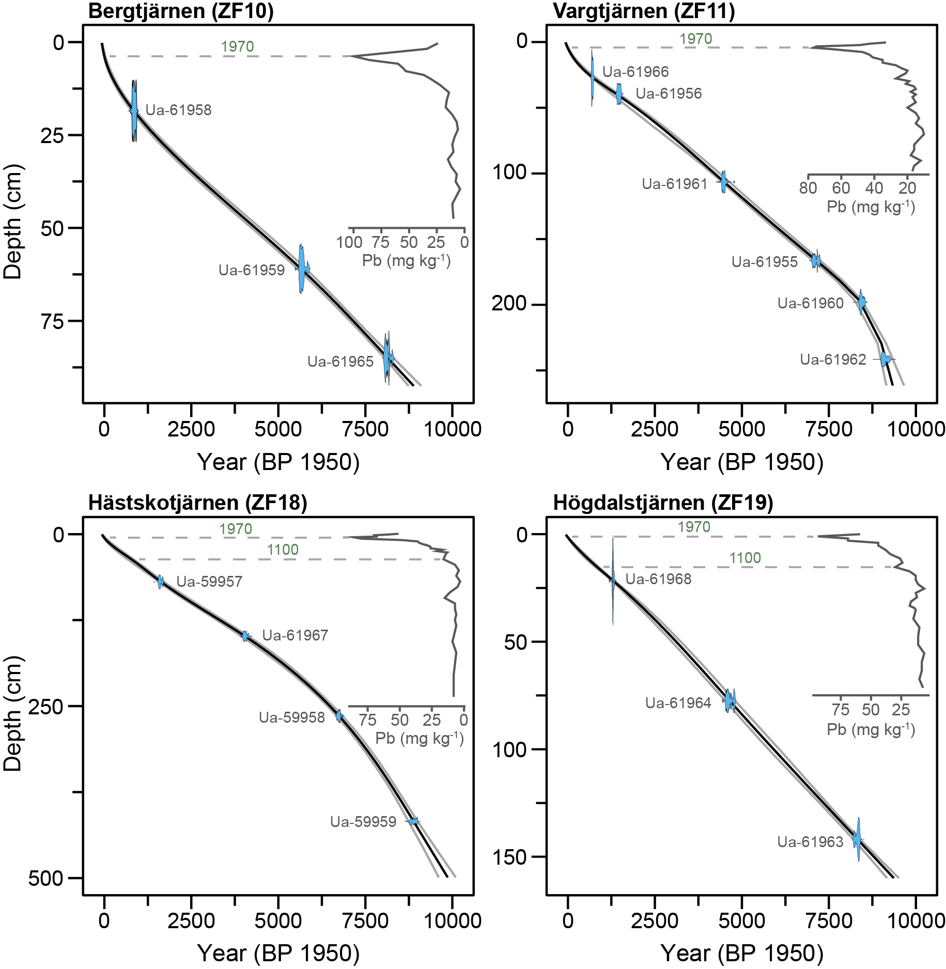

Supplement: Supplementary file 1 [file microorganisms-09-00355-s001.zip › microorganisms-1087079/Supplementary_Figure_1.tif]

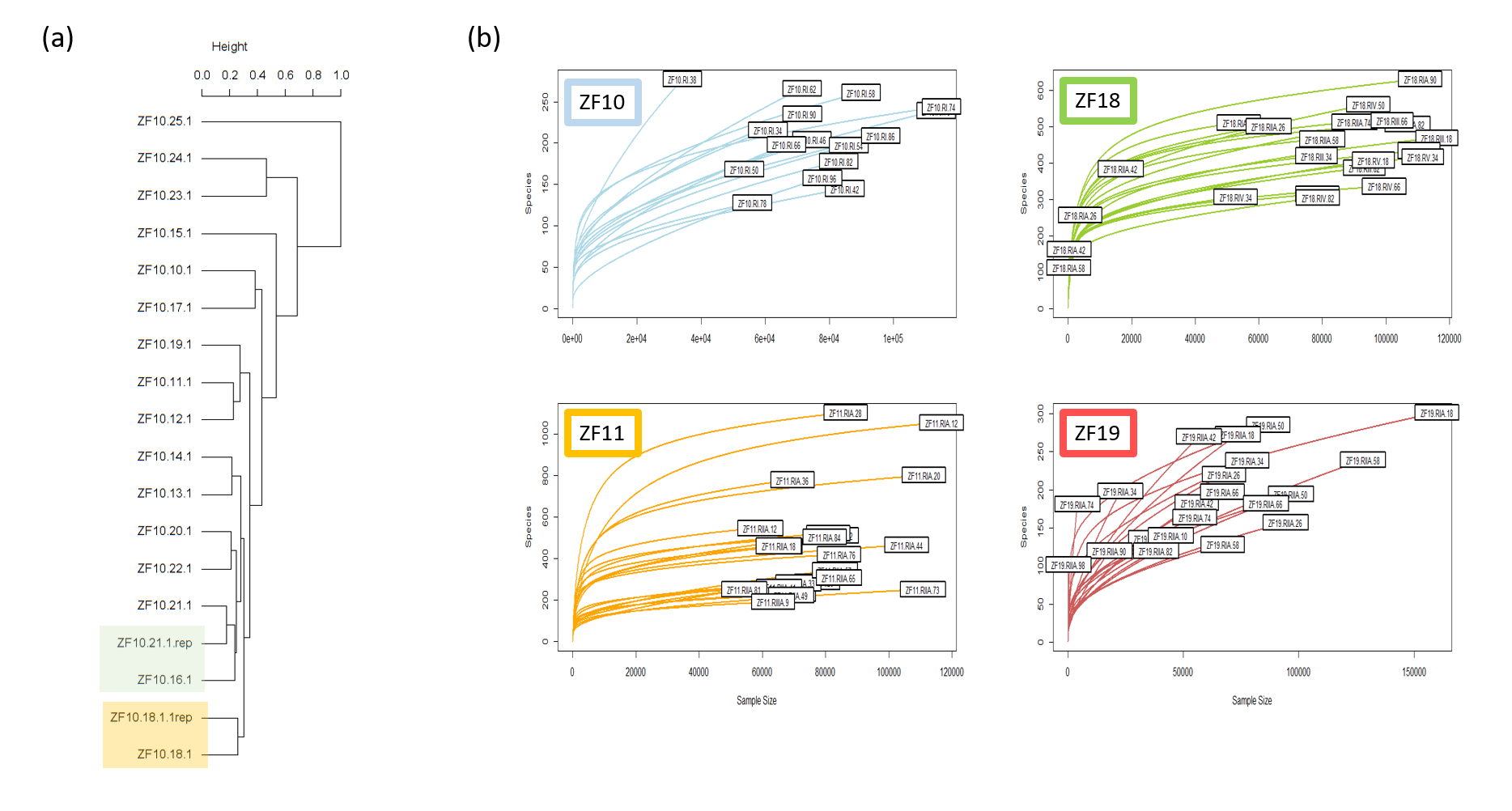

Supplement: Supplementary file 1 [file microorganisms-09-00355-s001.zip › microorganisms-1087079/Supplementary_Figure_2.tif]

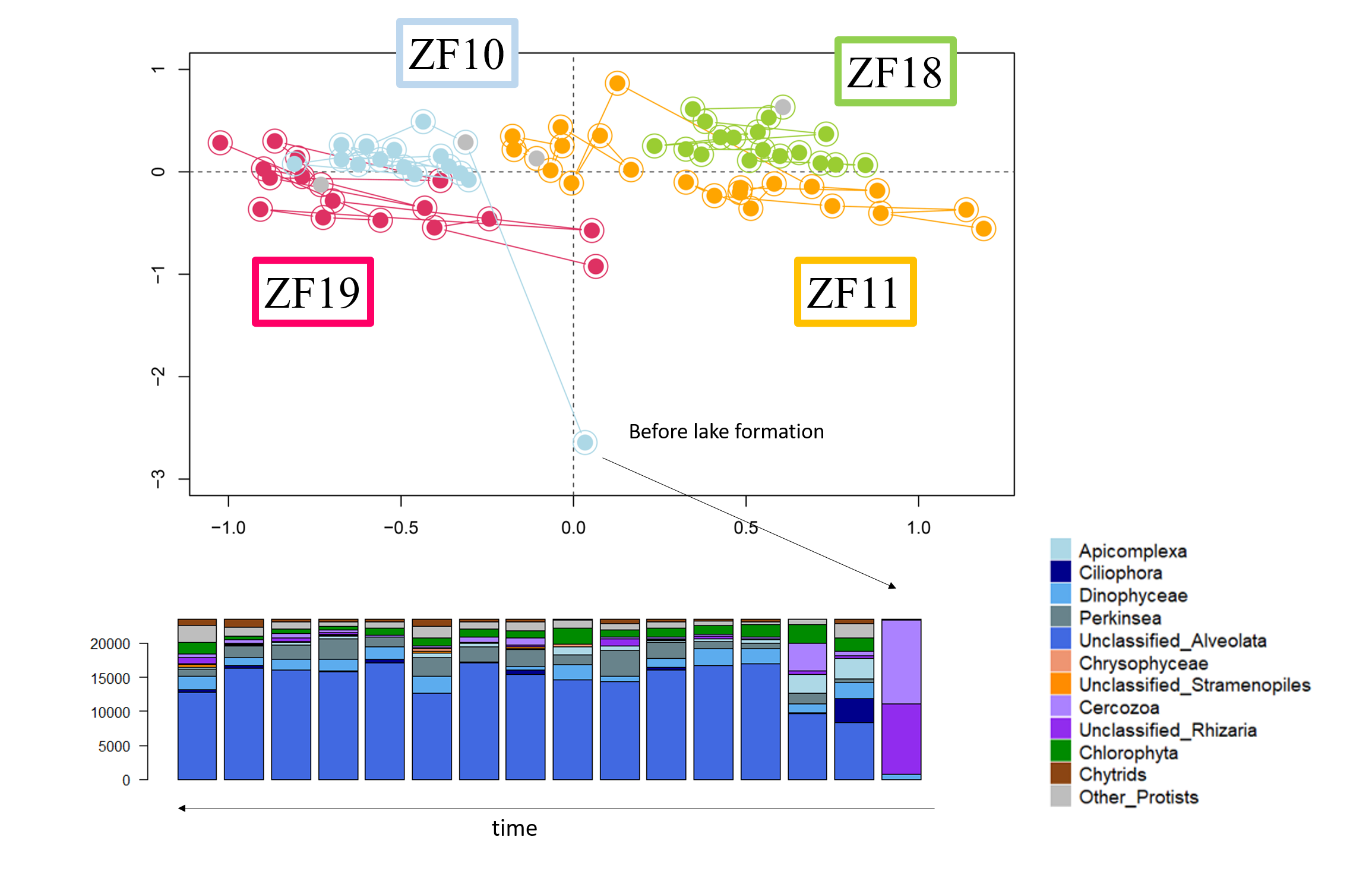

Supplement: Supplementary file 1 [file microorganisms-09-00355-s001.zip › microorganisms-1087079/Supplementary_Figure_3.tif]
